# Respiratory Syncytial Virus Seasonality — United States, 2014–2017

**DOI:** 10.15585/mmwr.mm6702a4

**Published:** 2018-01-19

**Authors:** Erica Billig Rose, Alexandra Wheatley, Gayle Langley, Susan Gerber, Amber Haynes

**Affiliations:** ^1^Epidemic Intelligence Service, Division of Scientific Education and Professional Development, CDC; ^2^Division of Viral Diseases, National Center for Immunizations and Respiratory Diseases, CDC.

Respiratory syncytial virus (RSV) is a leading cause of lower respiratory tract infection in young children worldwide (*1*–*3*). In the United States, RSV infection results in >57,000 hospitalizations and 2 million outpatient visits each year among children aged <5 years (*3*). Recent studies have highlighted the importance of RSV in adults as well as children (*4*). CDC reported RSV seasonality nationally, by U.S. Department of Health and Human Services (HHS) regions* and for the state of Florida, using a new statistical method that analyzes polymerase chain reaction (PCR) laboratory detections reported to the National Respiratory and Enteric Virus Surveillance System (NREVSS) (https://www.cdc.gov/surveillance/nrevss/index.html). Nationally, across three RSV seasons, lasting from the week ending July 5, 2014 through July 1, 2017, the median RSV onset occurred at week 41 (mid-October), and lasted 31 weeks until week 18 (early May). The median national peak occurred at week 5 (early February). Using these new methods, RSV season circulation patterns differed from those reported from previous seasons (*5*). Health care providers and public health officials use RSV circulation data to guide diagnostic testing and to time the administration of RSV immunoprophylaxis for populations at high risk for severe respiratory illness (*6*). With several vaccines and other immunoprophlyaxis products in development, estimates of RSV circulation are also important to the design of clinical trials and future vaccine effectiveness studies.

Participating clinical and public health laboratories voluntarily report the number of aggregate and positive RSV tests to NREVSS each week. In previous years, the RSV season was defined by consecutive weeks when RSV antigen-based tests exceeded 10% positivity (*5*); however, since 2008, laboratories have shifted away from antigen-based RSV testing, and since 2014 the majority of tests and RSV detections among consistently reporting laboratories are determined by PCR (*7*). From July through the following June of 2014–15, 2015–16 and 2016–17, approximately 56%, 62%, and 72% of RSV detections, respectively, were reported by PCR methods. To account for these observed changes in testing practice and to more accurately reflect recent circulation patterns, only results from PCR detection methods are included in this report.

The method that consistently captured the highest percentage of PCR detections for retrospectively characterizing RSV seasons was determined to be the retrospective slope 10 (RS10) method (*7*). This method uses a centered 5-week moving average of RSV detections normalized to a season peak of 1,000 detections. The season onset was defined as the second of 2 consecutive weeks when the slope, or normalized 5-week moving average of RSV detections between subsequent weeks, exceeded 10. The season offset was the last week when the standardized (normalized) detections exceeded the standardized detections at onset. The peak was the week with the most standardized detections. The season duration was the inclusive weeks between onset and offset.

Because patterns of weekly RSV circulation in Florida are different from regional and national patterns, Florida data are reported separately from other national data. RSV circulation patterns also appear to differ for Hawaii compared with other states in Region 9 based on limited antigen testing. Therefore, onset, offset, peak, and duration were summarized using the median of the three seasons nationally (with and without Florida and Hawaii), by HHS region, and for Florida. There are an insufficient number of Hawaii laboratories consistently reporting PCR data to present the state data separately with confidence. Laboratories were included in the analysis if they consistently conducted PCR testing, as defined by the following criteria: 1) reported RSV PCR testing results for ≥30 weeks during the 12-month NREVSS surveillance year and 2) averaged ≥10 PCR tests per week during the 52 weeks of the NREVSS season.^†^

From the week ending July 5, 2014 through July 1, 2017, there were three distinct RSV seasons: 2014–15, 2015–16, and 2016–17 (Figure). For each of these seasons, 135, 218, and 244 laboratories, respectively, reported at least 1 week of RSV testing by PCR to NREVSS. This analysis was limited to 80 (59%), 108 (50%), and 118 (48%) qualifying laboratories for 2014–15, 2015–16, and 2016–17, respectively (Table 1). The seasons as determined by the RS10 method captured 98% of reported RSV PCR detections during the 2014–15 reporting period and 97% of those reported during the 2015–16 and 2016–17 reporting periods.

**FIGURE:**
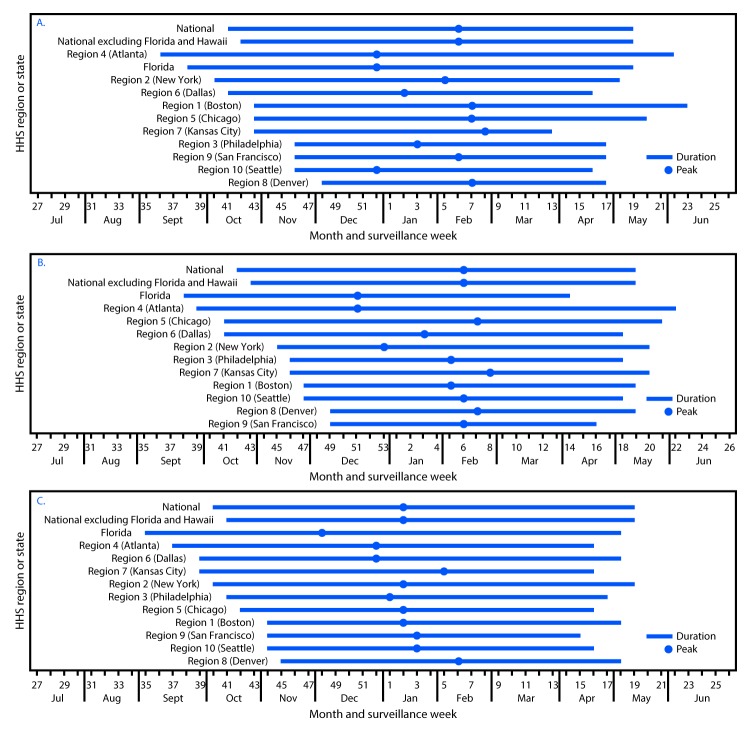
Respiratory syncytial virus season duration and peak, by U.S. Department of Health and Human Services (HHS**)** Region (headquarters),*^,†,§^ and in Florida — National Respiratory and Enteric Virus Surveillance System, United States, July 2014–June 2015 (A), July 2015–June 2016 (B), and July 2016–June 2017 (C) * Listed by region number and headquarters city. *Region 1 *(*Boston*): Connecticut, Maine, Massachusetts, New Hampshire, Rhode Island, and Vermont; *Region 2 *(*New York*): New Jersey and New York; *Region 3 *(*Philadelphia*): Delaware, District of Columbia, Maryland, Pennsylvania, Virginia, and West Virginia; *Region 4 *(*Atlanta*): Alabama, Florida, Georgia, Kentucky, Mississippi, North Carolina, South Carolina, and Tennessee; *Region 5 *(*Chicago*): Illinois, Indiana, Michigan, Minnesota, Ohio, and Wisconsin; *Region 6 *(*Dallas*): Arkansas, Louisiana, New Mexico, Oklahoma, and Texas; *Region 7 *(*Kansas City*): Iowa, Kansas, Missouri, and Nebraska; *Region 8 *(*Denver*): Colorado, Montana, North Dakota, South Dakota, Utah, and Wyoming; *Region 9 *(*San Francisco*): Arizona, California, Hawaii, and Nevada; *Region 10 *(*Seattle*): Alaska, Idaho, Oregon, and Washington. Delaware, District of Columbia, Idaho, Iowa, Maine, Maryland, Mississippi, Nebraska, New Hampshire, New Mexico, North Carolina, Rhode Island, Tennessee, and Wyoming did not have laboratories meeting the inclusion criteria for the 2014–15 season analysis. District of Columbia, Idaho, Maine, Mississippi, Nebraska, Nevada, New Hampshire, North Carolina, Rhode Island, Tennessee, and Wyoming did not have laboratories meeting the inclusion criteria for the 2015–16 season analysis. District of Columbia, Maine, Nevada, New Hampshire, Rhode Island, Tennessee, and Wyoming did not have laboratories meeting the inclusion criteria for the 2016–17 season analysis. ^†^ Region 4 (Atlanta) excludes data from Florida. ^§^ Region 9 (San Francisco) excludes data from Hawaii.

**TABLE 1:** Summary of 2014–15, 2015–16, and 2016–17 respiratory syncytial virus (RSV) seasons, by U.S. Departments of Health and Human Services (HHS) Region,* and in Florida — National Respiratory and Enteric Virus Surveillance System, July 2014–June 2017

HHS region (headquarters) or state/RSV season	No. of laboratories reporting	Onset week ending	Peak week ending	Offset week ending	Season duration (wks)
**National**					
2014–15^†^	80	10/11/2014	02/07/2015	05/09/2015	31
2015–16^§^	108	10/17/2015	02/13/2016	05/14/2016	31
2016–17^¶^	118	10/08/2016	01/14/2017	04/29/2017	30
**National without Florida and Hawaii**					
2014–15^†^	77	10/18/2014	02/07/2015	05/09/2015	30
2015–16^§^	104	10/24/2015	02/13/2016	05/14/2016	30
2016–17^¶^	113	10/15/2016	01/14/2017	04/29/2017	29
**Region 1 (Boston)**					
2014–15^†^	4	10/25/2014	02/14/2015	06/06/2015	33
2015–16^§^	5	11/21/2015	02/06/2016	05/14/2016	26
2016–17^¶^	7	11/05/2016	01/14/2017	05/06/2017	27
**Region 2 (New York)**					
2014–15^†^	6	10/04/2014	01/31/2015	05/02/2015	31
2015–16^§^	8	10/31/2015	01/02/2016	05/21/2016	30
2016–17^¶^	7	10/08/2016	01/14/2017	05/13/2017	32
**Region 3 (Philadelphia)**					
2014–15^†^	5	11/15/2014	01/10/2015	04/25/2015	24
2015–16^§^	10	11/07/2015	02/06/2016	05/07/2016	27
2016–17^¶^	9	10/15/2016	01/07/2017	04/29/2017	29
Region 4** (Atlanta)					
2014–15^†^	6	09/06/2014	12/27/2014	05/30/2015	39
2015–16^§^	7	09/26/2015	12/19/2015	06/04/2016	37
2016–17^¶^	7	09/17/2016	12/31/2016	04/22/2017	32
**Region 5 (Chicago)**					
2014–15^†^	22	10/25/2014	02/14/2015	05/16/2015	30
2015–16^§^	29	10/10/2015	02/20/2016	05/28/2016	34
2016–17^¶^	28	10/22/2016	01/14/2017	04/22/2017	27
**Region 6 (Dallas)**					
2014–15^†^	10	10/11/2014	01/10/2015	04/18/2015	28
2015–16^§^	11	10/10/2015	01/23/2016	05/07/2016	31
2016–17^¶^	14	10/01/2016	12/31/2016	05/06/2017	32
**Region 7 (Kansas City)**					
2014–15^†^	3	10/25/2014	02/21/2015	05/16/2015	30
2015–16^§^	5	11/14/2015	02/27/2016	05/21/2016	34
2016–17^¶^	7	10/01/2016	02/04/2017	04/22/2017	30
**Region 8 (Denver)**					
2014–15^†^	7	11/29/2014	02/14/2015	04/25/2015	22
2015–16^§^	10	12/05/2015	02/20/2016	05/14/2016	24
2016–17^¶^	11	11/12/2016	02/11/2017	05/06/2017	26
**Region 9^††^ (San Francisco)**					
2014–15^†^	9	11/15/2014	02/07/2015	04/11/2015	22
2015–16^§^	13	12/05/2015	02/13/2016	04/23/2016	21
2016–17^¶^	14	11/05/2016	01/21/2017	04/15/2017	24
**Region 10 (Seattle)**					
2014–15^†^	6	11/15/2014	01/31/2015	04/18/2015	23
2015–16^§^	7	11/21/2015	02/13/2016	05/07/2016	25
2016–17^¶^	10	11/05/2016	01/21/2017	04/22/2017	25
**Florida**					
2014–15^†^	2	09/20/2014	12/27/2014	05/09/2015	34
2015–16^§^	3	09/19/2015	12/19/2015	04/09/2016	30
2016–17^¶^	4	09/03/2016	12/03/2016	04/22/2017	34

* Listed by region number and headquarters city. *Region 1 *(*Boston*): Connecticut, Maine, Massachusetts, New Hampshire, Rhode Island, and Vermont; *Region 2 *(*New York*): New Jersey and New York; *Region 3 *(*Philadelphia*): Delaware, District of Columbia, Maryland, Pennsylvania, Virginia, and West Virginia; *Region 4 *(*Atlanta*): Alabama, Florida, Georgia, Kentucky, Mississippi, North Carolina, South Carolina, and Tennessee; *Region 5 *(*Chicago*): Illinois, Indiana, Michigan, Minnesota, Ohio, and Wisconsin; *Region 6 *(*Dallas*): Arkansas, Louisiana, New Mexico, Oklahoma, and Texas; *Region 7 *(*Kansas City*): Iowa, Kansas, Missouri, and Nebraska; *Region 8 *(*Denver*): Colorado, Montana, North Dakota, South Dakota, Utah, and Wyoming; *Region 9 *(*San Francisco*): Arizona, California, Hawaii, and Nevada; *Region 10 *(*Seattle*): Alaska, Idaho, Oregon, and Washington.

^†^ Delaware, District of Columbia, Idaho, Iowa, Maine, Maryland, Mississippi, Nebraska, New Hampshire, New Mexico, North Carolina, Rhode Island, Tennessee, and Wyoming did not have laboratories meeting the inclusion criteria for the 2014–15 season analysis.

^§^ District of Columbia, Idaho, Maine, Mississippi, Nebraska, Nevada, New Hampshire, North Carolina, Rhode Island, Tennessee, and Wyoming did not have laboratories meeting the inclusion criteria for the 2015–16 season analysis.

^¶^ District of Columbia, Maine, Nevada, New Hampshire, Rhode Island, Tennessee, and Wyoming did not have laboratories meeting the inclusion criteria for the 2016–17 season analysis.

** Excludes data from Florida.

^††^ Excludes data from Hawaii.

Nationally, across the three seasons, the median RSV onset occurred at surveillance week 41 (mid-October), and lasted 31 weeks until surveillance week 18 (early May) (Table 2). The median national peak occurred at week 5 (early February). When Florida and Hawaii are excluded, the national onset occurred 1 week later and the season duration decreased by 1 week. Median onset for the 10 HHS regions (excluding Florida and Hawaii) ranged from week 37 to week 48 (mid-September to early December) and offset ranged from week 15 to week 21 (mid-April to late May) (Figure). The median season peaks ranged from week 52 to week 7 (late December to mid-February), and the median duration ranged from 22 to 37 weeks (Table 2). Region 9 had the shortest season (median = 22 weeks), and Region 4 had the longest (37 weeks). The median onset for Florida occurred at week 37 (mid-September), and the season continued through week 16 (mid-April) (Table 2).

**TABLE 2:** Summary of 2014–15, 2015–16, and 2016–17 respiratory syncytial virus seasons by median and range, by U.S. Departments of Health and Human Services (HHS) Region,* and in Florida — National Respiratory and Enteric Virus Surveillance System, July 2014–June 2017

HHS region or state	2014–2017 season median and range (surveillance week number)							
	Onset median surveillance week (mo)	Onset range surveillance weeks (mos)	Peak median surveillance week (mo)	Peak range surveillance weeks (mos)	Offset median surveillance week (mo)	Offset range surveillance weeks (mos)	Median duration (wks)	Duration range (wks)
**National**	**41 (mid-Oct)**	**40–41 (Oct)**	**5 (early Feb)**	**2–6 (Jan–Feb)**	**18 (early May)**	**17–19 (Apr–May)**	**31**	**30–31**
National (excluding Florida and Hawaii)	42 (mid-Oct)	41–42 (Oct)	5 (early Feb)	2–6 (Jan–Feb)	18 (early May)	17–19 (Apr–May)	30	29–30
Region 1	44 (late Oct)	43–46 (Oct–Nov)	5 (early Feb)	2–6 (Jan–Feb)	19 (mid-May)	18–22 (May)	27	26–33
Region 2	40 (early Oct)	40–43 (Oct)	2 (mid-Jan)	52–4 (Dec–Jan)	19 (mid-May)	17–20 (Apr–May)	31	30–32
Region 3	44 (late Oct)	41–46 (Oct–Nov)	1 (mid-Jan)	1–5 (Jan–Feb)	17 (late Apr)	16–18 (Apr–May)	27	24–29
Region 4^†^	37 (mid-Sep)	36–38 (Sep)	52 (late Dec)	50–52 (Dec)	21 (late May)	16–22 (Apr–May)	37	32–39
Region 5	42 (mid-Oct)	40–43 (Oct)	6 (mid-Feb)	2–7 (Jan–Feb)	19 (mid-May)	16–21 (Apr–May)	30	27–34
Region 6	40 (early Oct)	39–41 (Sep–Oct)	1 (mid-Jan)	52–3 (Dec–Jan)	18 (early May)	15–18 (Apr–May)	31	28–32
Region 7	43 (late Oct)	39–45 (Sep–Nov)	7 (mid-Feb)	5–8 (Feb)	19 (mid-May)	16–20 (Apr–May)	30	30–34
Region 8	48 (late Nov)	45–48 (Nov)	6 (mid-Feb)	6–7 (Feb)	18 (early May)	16–19 (Apr–May)	24	22–26
Region 9^§^	46 (mid-Nov)	44–48 (Nov)	5 (early Feb)	3–6 (Jan–Feb)	15 (mid-Apr)	14–16 (Apr)	22	21–24
Region 10	46 (mid-Nov)	44–46 (Nov)	4 (late Jan)	3–6 (Jan–Feb)	16 (mid-Apr)	15–18 (Apr–May)	25	23–25
Florida	37 (mid-Sep)	35–38 (Aug–Sep)	50 (mid-Dec)	48–52 (Dec)	16 (mid-Apr)	14–18 (Apr–May)	34	30–34

* Listed by region number and headquarters city. *Region 1 *(*Boston*): Connecticut, Maine, Massachusetts, New Hampshire, Rhode Island, and Vermont; *Region 2 *(*New York*): New Jersey and New York; *Region 3 *(*Philadelphia*): Delaware, District of Columbia, Maryland, Pennsylvania, Virginia, and West Virginia; *Region 4 *(*Atlanta*): Alabama, Florida, Georgia, Kentucky, Mississippi, North Carolina, South Carolina, and Tennessee; *Region 5 *(*Chicago*): Illinois, Indiana, Michigan, Minnesota, Ohio, and Wisconsin; *Region 6 *(*Dallas*): Arkansas, Louisiana, New Mexico, Oklahoma, and Texas; *Region 7 *(*Kansas City*): Iowa, Kansas, Missouri, and Nebraska; *Region 8 *(*Denver*): Colorado, Montana, North Dakota, South Dakota, Utah, and Wyoming; *Region 9 *(*San Francisco*): Arizona, California, Hawaii, and Nevada; *Region 10 *(*Seattle*): Alaska, Idaho, Oregon, and Washington. Delaware, District of Columbia, Idaho, Iowa, Maine, Maryland, Mississippi, Nebraska, New Hampshire, New Mexico, North Carolina, Rhode Island, Tennessee, and Wyoming did not have laboratories meeting the inclusion criteria for the 2014–15 season analysis. District of Columbia, Idaho, Maine, Mississippi, Nebraska, Nevada, New Hampshire, North Carolina, Rhode Island, Tennessee, and Wyoming did not have laboratories meeting the inclusion criteria for the 2015–16 season analysis. District of Columbia, Maine, Nevada, New Hampshire, Rhode Island, Tennessee, and Wyoming did not have laboratories meeting the inclusion criteria for the 2016–17 season analysis.

^†^ Excludes data from Florida.

^§^ Excludes data from Hawaii.

## Discussion

The national RSV season onsets and offsets reported here occurred in different surveillance weeks than those reported in previous seasons (*5*). Using PCR data reported to NREVSS, onsets for the 2014–15, 2015–16, and 2016–17 seasons occurred approximately 2 weeks earlier than did those for the 2012–13 and 2013–2014 seasons (early to mid-October versus late October to early November), which were determined using antigen data; similarly, offsets occurred approximately 4 weeks later (late April to early May versus late March). These differences largely reflect the adoption of a statistical method that identifies a consistent inflection point in weekly RSV detections, rather than a threshold of weekly positivity influenced heavily by the volume of tests performed (*7*). The differences inherent in evaluating PCR tests, many of which detect several viral respiratory pathogens, compared with RSV antigen tests, that exclusively detect RSV, necessitated the adoption of a new statistical method to capture a consistently high proportion of RSV detections within the defined season (*7*). This change in methodology has resulted in a relative lengthening of the RSV seasons.

Using antigen-based methods, in past years Florida has been observed to have an earlier onset than other states in the country (*8*). However, using the RS10 method, this earlier onset was not consistently observed. This report included fewer consistently reporting laboratories in Florida compared with previous seasons, and the observed patterns might not represent the entire state. Previous limited antigen-based testing shows that seasonality in Hawaii might differ from that in other states in Region 9, but too few laboratories have consistently reported PCR data during the analysis period to present these data separately (https://www.cdc.gov/surveillance/nrevss/rsv/state.html#HI). Many factors might influence national, regional, and county-level RSV activity, including social and demographic factors, population density, pollution, and climate (*8*–*10*).

NREVSS surveillance data reflect recent circulation patterns of RSV and might inform policy decisions regarding administration of palivizumab for immunoprophylaxis. Palivizumab is a monoclonal antibody recommended by the American Academy of Pediatrics for administration during the RSV season to infants at high risk and young children likely to benefit from immunoprophylaxis, based on their gestational age at birth and the presence of certain underlying medical conditions during the RSV season (*6*).^§^ In addition, RSV seasonality data might inform the timing of clinical trials for several RSV vaccines and immunoprophlyaxis products in development, as well as the evaluation of product effectiveness after licensure. As testing methods and practices continue to evolve, CDC might further refine the approach to ascertaining RSV seasons.

The findings in this report are subject to at least four limitations. First, reporting to NREVSS is voluntary, and analysis is limited to consistently reporting laboratories, which might not fully represent local and regional circulation. Second, low RSV circulation might not be captured within the NREVSS onset and offset, although at least 97% of detections were accounted for using the RS10 method. Third, this report only includes PCR detections. Although this represents a majority of detections among consistent reporters, 28%–44% of detections are by antigen methods. Finally, although the number of positive detections is dependent upon the number of tests ordered, the RS10 method minimizes this bias by normalizing the detections. Despite these limitations, NREVSS provides useful information to clinicians regarding RSV circulation and to researchers designing clinical trials for vaccines and immunoprophylaxis products under development.

The RS10 method used here captures a high proportion of RSV PCR detections for retrospectively determining RSV seasonality, but cannot be used to determine seasonal onset and offset in real time, and can only be employed after the season ends. Alternative statistical methods, including the tenfold baseline or 3% threshold methods (*7*) might be used to determine seasonality in real time or near real time. Timely NREVSS data and updates of RSV activity at the national, regional, and state levels are published online weekly at https://www.cdc.gov/surveillance/nrevss. Surveillance data collected by state and local health departments might more accurately describe local RSV circulation trends.

**Summary**

**What is already known about this topic?**

For most of the United States, the respiratory syncytial virus (RSV) season lasts from fall through spring but varies from year to year and by geographic region.

**What is added by this report?**

This report uses a new statistical method that analyzes polymerase chain reaction laboratory detections reported to the National Respiratory and Enteric Virus Surveillance System (NREVSS) to determine RSV seasonality nationally and by region for three recent seasons (2014–2017). Nationally, lasting from the week ending July 5, 2014 through July 1, 2017, the median RSV onset occurred at week 41 (mid-October), and lasted 31 weeks until week 18 (early May). The median national peak occurred at week 5 (early February). Onsets for the 2014–17 seasons occurred approximately 2 weeks earlier than did those for the 2012–2014 seasons (early to mid-October versus late October to early November), which were determined using antigen data.

**What are the implications for public health practice?**

RSV seasonality data can guide diagnostic testing and inform policy decisions regarding administration of currently available immunoprophylaxis products, when indicated, and the timing of clinical trials and future evaluations of vaccines and immunoprophylaxis products currently under development.

## Footnotes

* Listed by region number and headquarters city. *Region 1 *(*Boston*): Connecticut, Maine, Massachusetts, New Hampshire, Rhode Island, and Vermont; *Region 2 *(*New York*): New Jersey and New York; *Region 3 *(*Philadelphia*): Delaware, District of Columbia, Maryland, Pennsylvania, Virginia, and West Virginia; *Region 4 *(*Atlanta*): Alabama, Florida, Georgia, Kentucky, Mississippi, North Carolina, South Carolina, and Tennessee; *Region 5 *(*Chicago*): Illinois, Indiana, Michigan, Minnesota, Ohio, and Wisconsin; *Region 6 *(*Dallas*): Arkansas, Louisiana, New Mexico, Oklahoma, and Texas; *Region 7 *(*Kansas City*): Iowa, Kansas, Missouri, and Nebraska; *Region 8 *(*Denver*): Colorado, Montana, North Dakota, South Dakota, Utah, and Wyoming; *Region 9 *(*San Francisco*): Arizona, California, Hawaii, and Nevada; *Region 10 *(*Seattle*): Alaska, Idaho, Oregon, and Washington. Delaware, District of Columbia, Idaho, Iowa, Maine, Maryland, Mississippi, Nebraska, New Hampshire, New Mexico, North Carolina, Rhode Island, Tennessee, and Wyoming did not have laboratories meeting the inclusion criteria for the 2014–15 season analysis. District of Columbia, Idaho, Maine, Mississippi, Nebraska, Nevada, New Hampshire, North Carolina, Rhode Island, Tennessee, and Wyoming did not have laboratories meeting the inclusion criteria for the 2015–16 season analysis. District of Columbia, Maine, Nevada, New Hampshire, Rhode Island, Tennessee, and Wyoming did not have laboratories meeting the inclusion criteria for the 2016–17 season analysis.

† The 12 months included in a particular reporting season run from July through June.

§ CDC does not make recommendations regarding the administration of RSV immunoprophylaxis.

## References

[1] Shi T, McAllister DA, O’Brien KL, Global, regional, and national disease burden estimates of acute lower respiratory infections due to respiratory syncytial virus in young children in 2015: a systematic review and modelling study. Lancet 2017;390:946–58. 28689664 10.1016/S0140-6736(17)30938-8 PMC5592248

[2] Hall CB, Weinberg GA, Blumkin AK, Respiratory syncytial virus-associated hospitalizations among children less than 24 months of age. Pediatrics 2013;132:e341–8. 23878043 10.1542/peds.2013-0303

[3] Hall CB, Weinberg GA, Iwane MK, The burden of respiratory syncytial virus infection in young children. N Engl J Med 2009;360:688–98. 10.1056/NEJMoa0804877 PMC4829966 19196675

[4] Falsey AR, Hennessey PA, Formica MA, Cox C, Walsh EE. Respiratory syncytial virus infection in elderly and high-risk adults. N Engl J Med 2005;352:1749–59. 15858184 10.1056/NEJMoa043951

[5] Haynes AK, Prill MM, Iwane MK, Gerber SI. Respiratory syncytial virus—United States, July 2012–June 2014. MMWR Morb Mortal Wkly Rep 2014;63:1133–6. 25474034 PMC4584603

[6] American Academy of Pediatrics Committee on Infectious Diseases, American Academy of Pediatrics Bronchiolitis Guidelines Committee. Updated guidance for palivizumab prophylaxis among infants and young children at increased risk of hospitalization for respiratory syncytial virus infection. Pediatrics 2014;134:415–20. 25070315 10.1542/peds.2014-1665

[7] Midgley CM, Haynes AK, Baumgardner JL, Determining the seasonality of respiratory syncytial virus in the United States: the impact of increased molecular testing. J Infect Dis 2017;216:345–55. 28859428 10.1093/infdis/jix275 PMC5712458

[8] Mullins JA, Lamonte AC, Bresee JS, Anderson LJ. Substantial variability in community respiratory syncytial virus season timing. Pediatr Infect Dis J 2003;22:857–62. 14551484 10.1097/01.inf.0000090921.21313.d3

[9] Zachariah P, Shah S, Gao D, Simoes EA. Predictors of the duration of the respiratory syncytial virus season. Pediatr Infect Dis J 2009;28:772–6. 19710584 10.1097/INF.0b013e3181a3e5b6

[10] Panozzo CA, Fowlkes AL, Anderson LJ. Variation in timing of respiratory syncytial virus outbreaks: lessons from national surveillance. Pediatr Infect Dis J 2007;26:S41–5. 18090199 10.1097/INF.0b013e318157da82

